# Supplementary material for: A scalable approach to resolving variants of uncertain significance
Source: bioRxiv. 2026 Feb 23:2026.02.14.705848. Originally published 2026 Feb 14. Preprint. [Version 2] doi: 10.64898/2026.02.14.705848 (PMC12918978; doi:10.64898/2026.02.14.705848)
Supplement: Supplement 9 [file media-9.pdf]

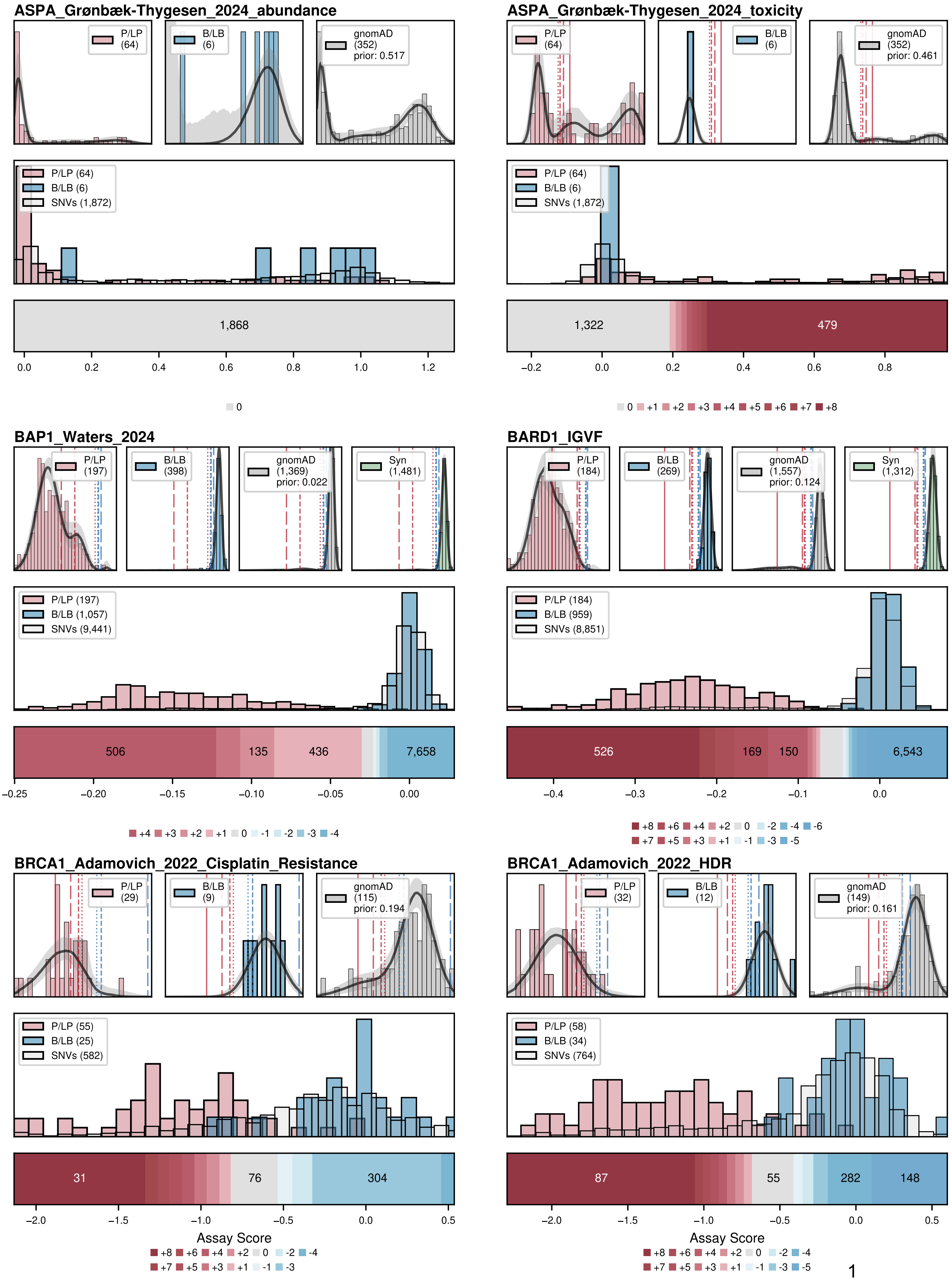
ASPA_Grønbæk-Thygesen_2024_abundance
P/LP (64)
B/LB (6)
gnomAD (352) prior: 0.517
P/LP (64)
B/LB (6)
SNVs (1,872)
1,868
0.0 0.2 0.4 0.6 0.8 1.0 1.2
0
ASPA_Grønbæk-Thygesen_2024_toxicity
P/LP (64)
B/LB (6)
gnomAD (352) prior: 0.461
P/LP (64)
B/LB (6)
SNVs (1,872)
1,322
479
−0.2 0.0 0.2 0.4 0.6 0.8
0 +1 +2 +3 +4 +5 +6 +7 +8
BAP1_Waters_2024
P/LP (197)
B/LB (398)
gnomAD (1,369) prior: 0.022
Syn (1,481)
P/LP (197)
B/LB (1,057)
SNVs (9,441)
506
135
436
7,658
−0.25 −0.20 −0.15 −0.10 −0.05 0.00
+4 +3 +2 +1 0 -1 -2 -3 -4
BARD1_IGVF
P/LP (184)
B/LB (269)
gnomAD (1,557) prior: 0.124
Syn (1,312)
P/LP (184)
B/LB (959)
SNVs (8,851)
526
169
150
6,543
−0.4 −0.3 −0.2 −0.1 0.0
+8 +6 +4 +2 0 -2 -4 -6
+7 +5 +3 +1 -1 -3 -5
BRCA1_Adamovich_2022_Cisplatin_Resistance
P/LP (29)
B/LB (9)
gnomAD (115) prior: 0.194
P/LP (55)
B/LB (25)
SNVs (582)
31
76
304
−2.0 −1.5 −1.0 −0.5 0.0 0.5
Assay Score
+8 +6 +4 +2 0 -2 -4
+7 +5 +3 +1 -1 -3
BRCA1_Adamovich_2022_HDR
P/LP (32)
B/LB (12)
gnomAD (149) prior: 0.161
P/LP (58)
B/LB (34)
SNVs (764)
87
55
282
148
−2.0 −1.5 −1.0 −0.5 0.0 0.5
Assay Score
+8 +6 +4 +2 0 -2 -4
+7 +5 +3 +1 -1 -3 -5

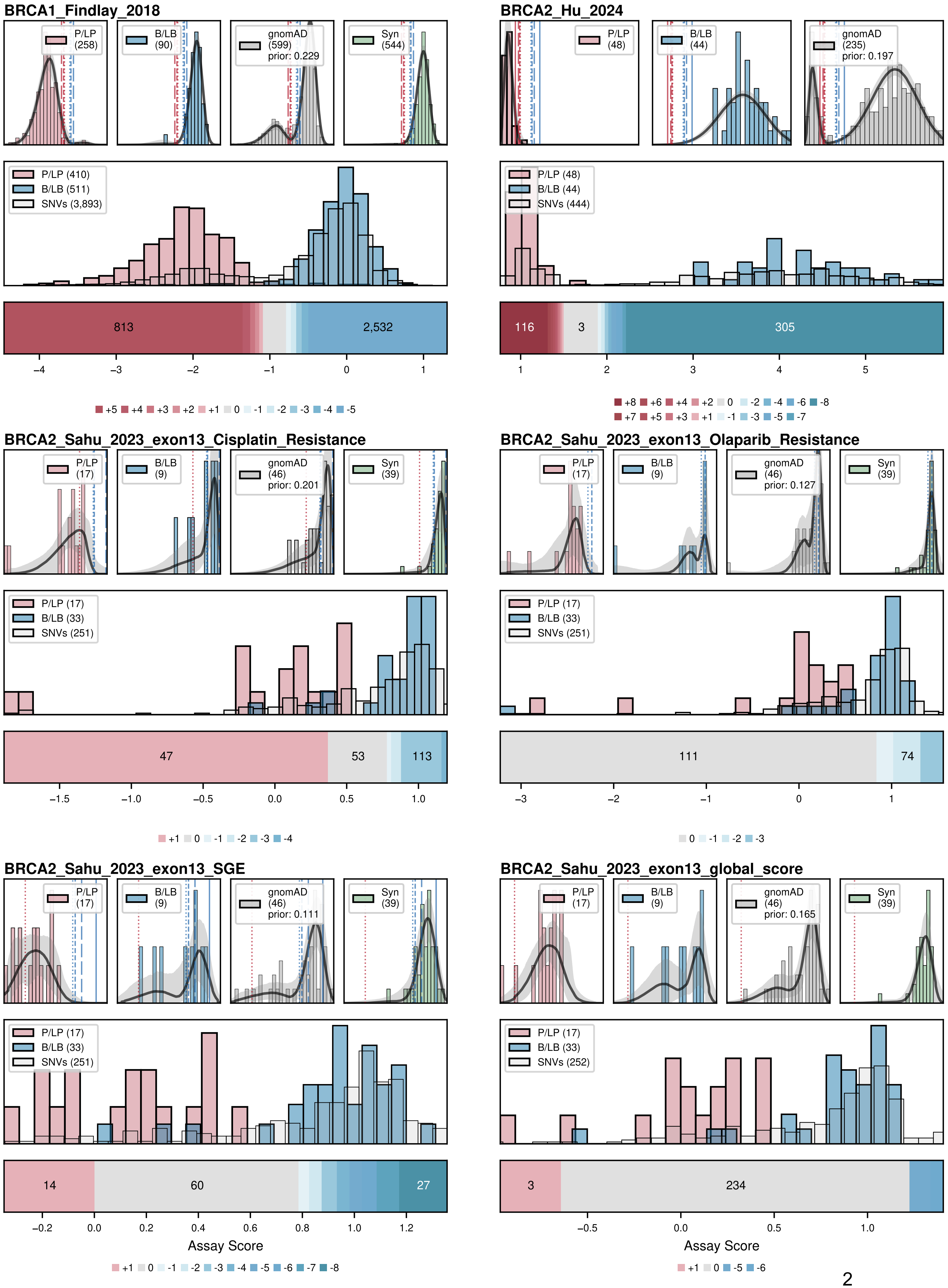
BRCA1_Findlay_2018
P/LP (258)
B/LB (90)
gnomAD (599) prior: 0.229
Syn (544)
P/LP (410)
B/LB (511)
SNVs (3,893)
813
2,532
−4 −3 −2 −1 0 1
+5 +4 +3 +2 +1 0 -1 -2 -3 -4 -5
BRCA2_Hu_2024
P/LP (48)
B/LB (44)
gnomAD (235) prior: 0.197
P/LP (48)
B/LB (44)
SNVs (444)
116
3
305
1 2 3 4 5
+8 +6 +4 +2 0 -2 -4 -6 -8
+7 +5 +3 +1 -1 -3 -5 -7
BRCA2_Sahu_2023_exon13_Cisplatin_Resistance
P/LP (17)
B/LB (9)
gnomAD (46) prior: 0.201
Syn (39)
P/LP (17)
B/LB (33)
SNVs (251)
47
53
113
−1.5 −1.0 −0.5 0.0 0.5 1.0
+1 0 -1 -2 -3 -4
BRCA2_Sahu_2023_exon13_Olaparib_Resistance
P/LP (17)
B/LB (9)
gnomAD (46) prior: 0.127
Syn (39)
P/LP (17)
B/LB (33)
SNVs (251)
111
74
−3 −2 −1 0 1
0 -1 -2 -3
BRCA2_Sahu_2023_exon13_SGE
P/LP (17)
B/LB (9)
gnomAD (46) prior: 0.111
Syn (39)
P/LP (17)
B/LB (33)
SNVs (251)
14
60
27
−0.2 0.0 0.2 0.4 0.6 0.8 1.0 1.2
Assay Score
+1 0 -1 -2 -3 -4 -5 -6 -7 -8
BRCA2_Sahu_2023_exon13_global_score
P/LP (17)
B/LB (9)
gnomAD (46) prior: 0.165
Syn (39)
P/LP (17)
B/LB (33)
SNVs (252)
3
234
−0.5 0.0 0.5 1.0
Assay Score
+1 0 -5 -6

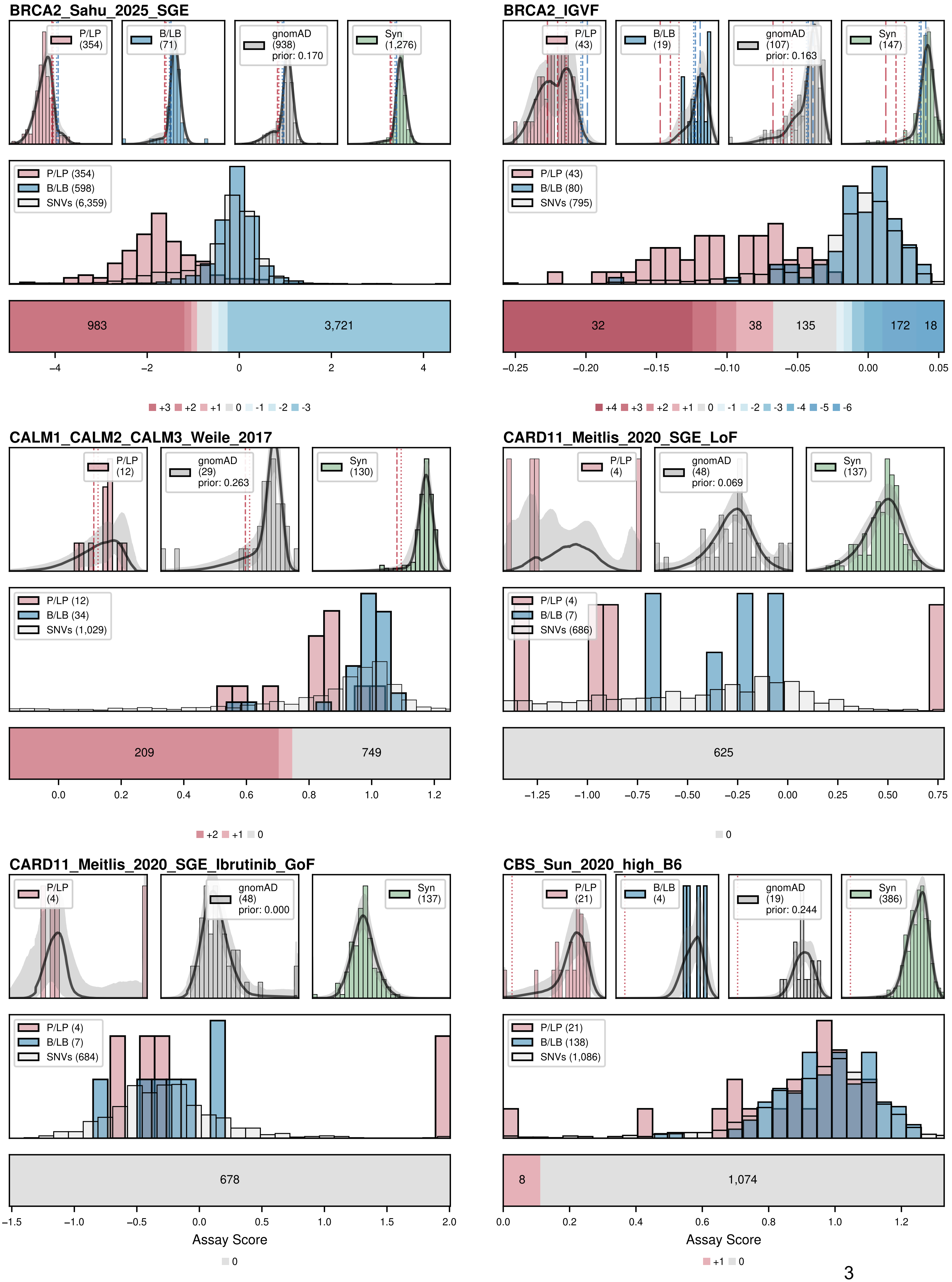
BRCA2_Sahu_2025_SGE
P/LP (354)
B/LB (71)
gnomAD (938) prior: 0.170
Syn (1,276)
P/LP (354)
B/LB (598)
SNVs (6,359)
983
3,721
+3 +2 +1 0 -1 -2 -3
BRCA2_IGVF
P/LP (43)
B/LB (19)
gnomAD (107) prior: 0.163
Syn (147)
P/LP (43)
B/LB (80)
SNVs (795)
32
38
135
172
18
+4 +3 +2 +1 0 -1 -2 -3 -4 -5 -6
CALM1_CALM2_CALM3_Weile_2017
P/LP (12)
gnomAD (29) prior: 0.263
Syn (130)
P/LP (12)
B/LB (34)
SNVs (1,029)
209
749
+2 +1 0
CARD11_Meitlis_2020_SGE_LoF
P/LP (4)
gnomAD (48) prior: 0.069
Syn (137)
P/LP (4)
B/LB (7)
SNVs (686)
625
0
CARD11_Meitlis_2020_SGE_Ibrutinib_GoF
P/LP (4)
gnomAD (48) prior: 0.000
Syn (137)
P/LP (4)
B/LB (7)
SNVs (684)
678
Assay Score
0
CBS_Sun_2020_high_B6
P/LP (21)
B/LB (4)
gnomAD (19) prior: 0.244
Syn (386)
P/LP (21)
B/LB (138)
SNVs (1,086)
8
1,074
Assay Score
+1 0

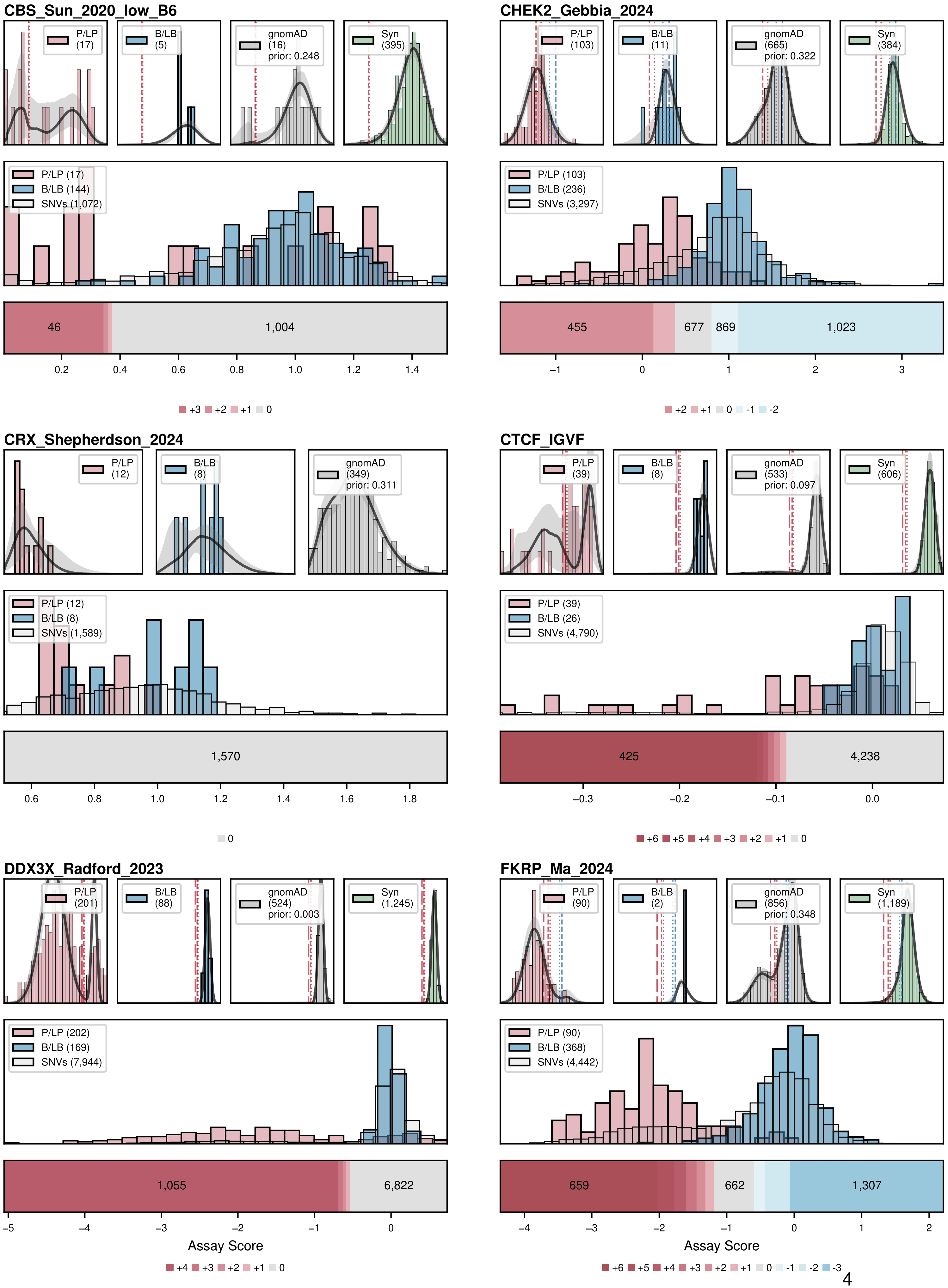
CBS_Sun_2020_low_B6
P/LP (17)
B/LB (5)
gnomAD (16) prior: 0.248
Syn (395)
P/LP (17)
B/LB (144)
SNVs (1,072)
46
1,004
0.2 0.4 0.6 0.8 1.0 1.2 1.4
+3 +2 +1 0
CHEK2_Gebbia_2024
P/LP (103)
B/LB (11)
gnomAD (665) prior: 0.322
Syn (384)
P/LP (103)
B/LB (236)
SNVs (3,297)
455
677
869
1,023
−1 0 1 2 3
+2 +1 0 -1 -2
CRX_Shepherdson_2024
P/LP (12)
B/LB (8)
gnomAD (349) prior: 0.311
P/LP (12)
B/LB (8)
SNVs (1,589)
1,570
0.6 0.8 1.0 1.2 1.4 1.6 1.8
0
CTCF_IGVF
P/LP (39)
B/LB (8)
gnomAD (533) prior: 0.097
Syn (606)
P/LP (39)
B/LB (26)
SNVs (4,790)
425
4,238
−0.3 −0.2 −0.1 0.0
+6 +5 +4 +3 +2 +1 0
DDX3X_Radford_2023
P/LP (201)
B/LB (88)
gnomAD (524) prior: 0.003
Syn (1,245)
P/LP (202)
B/LB (169)
SNVs (7,944)
1,055
6,822
−5 −4 −3 −2 −1 0
Assay Score
+4 +3 +2 +1 0
FKRP_Ma_2024
P/LP (90)
B/LB (2)
gnomAD (856) prior: 0.348
Syn (1,189)
P/LP (90)
B/LB (368)
SNVs (4,442)
659
662
1,307
−4 −3 −2 −1 0 1 2
Assay Score
+6 +5 +4 +3 +2 +1 0 -1 -2 -3

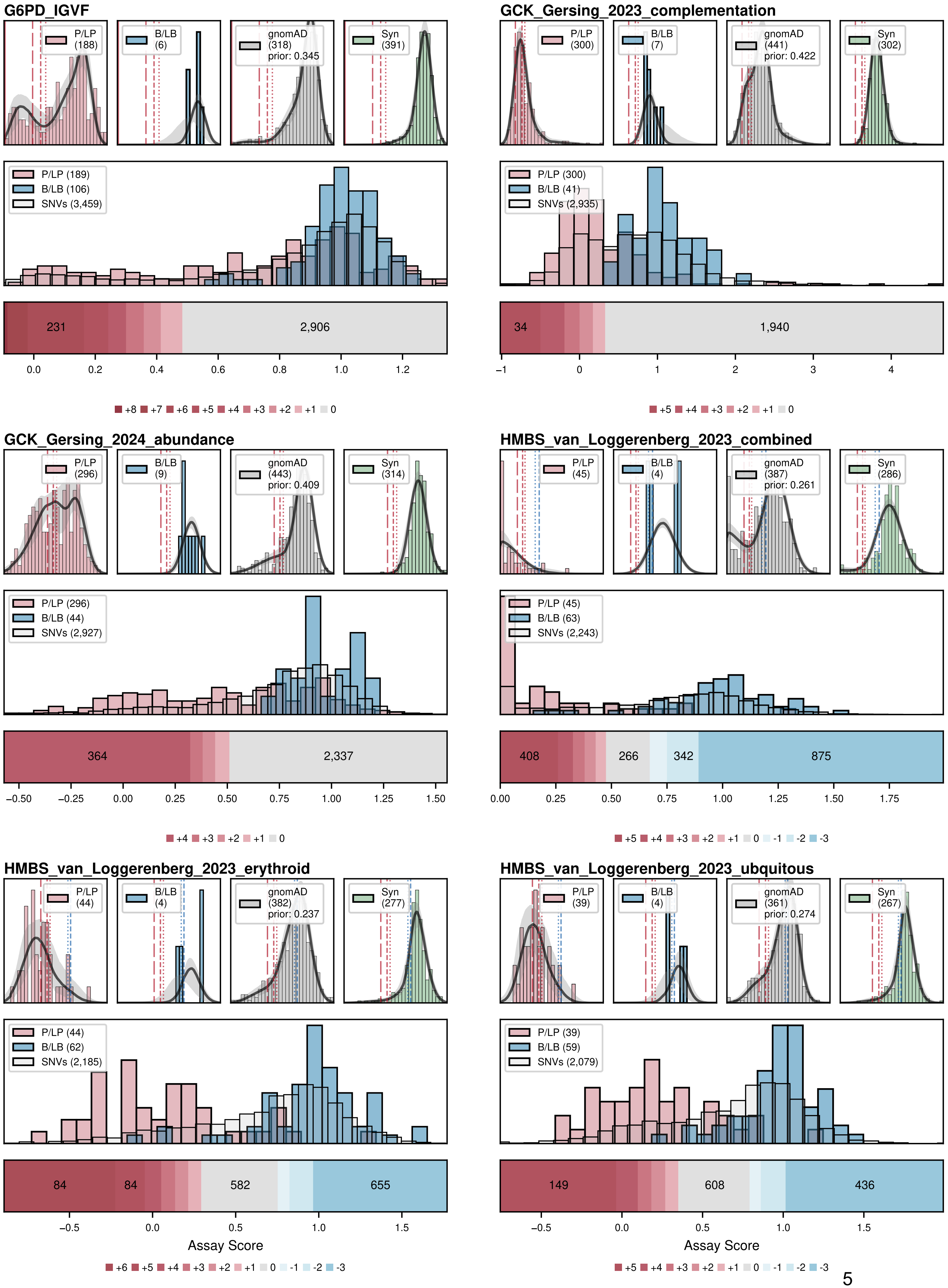
G6PD_IGVF
P/LP (188)
B/LB (6)
gnomAD (318) prior: 0.345
Syn (391)
P/LP (189)
B/LB (106)
SNVs (3,459)
231
2,906
0.0 0.2 0.4 0.6 0.8 1.0 1.2
+8 +7 +6 +5 +4 +3 +2 +1 0
GCK_Gersing_2023_complementation
P/LP (300)
B/LB (7)
gnomAD (441) prior: 0.422
Syn (302)
P/LP (300)
B/LB (41)
SNVs (2,935)
34
1,940
−1 0 1 2 3 4
+5 +4 +3 +2 +1 0
GCK_Gersing_2024_abundance
P/LP (296)
B/LB (9)
gnomAD (443) prior: 0.409
Syn (314)
P/LP (296)
B/LB (44)
SNVs (2,927)
364
2,337
−0.50 −0.25 0.00 0.25 0.50 0.75 1.00 1.25 1.50
+4 +3 +2 +1 0
HMBS_van_Loggerenberg_2023_combined
P/LP (45)
B/LB (4)
gnomAD (387) prior: 0.261
Syn (286)
P/LP (45)
B/LB (63)
SNVs (2,243)
408
266
342
875
0.00 0.25 0.50 0.75 1.00 1.25 1.50 1.75
+5 +4 +3 +2 +1 0 -1 -2 -3
HMBS_van_Loggerenberg_2023_erythroid
P/LP (44)
B/LB (4)
gnomAD (382) prior: 0.237
Syn (277)
P/LP (44)
B/LB (62)
SNVs (2,185)
84
84
582
655
−0.5 0.0 0.5 1.0 1.5
Assay Score
+6 +5 +4 +3 +2 +1 0 -1 -2 -3
HMBS_van_Loggerenberg_2023_ubquitous
P/LP (39)
B/LB (4)
gnomAD (361) prior: 0.274
Syn (267)
P/LP (39)
B/LB (59)
SNVs (2,079)
149
608
436
−0.5 0.0 0.5 1.0 1.5
Assay Score
+5 +4 +3 +2 +1 0 -1 -2 -3

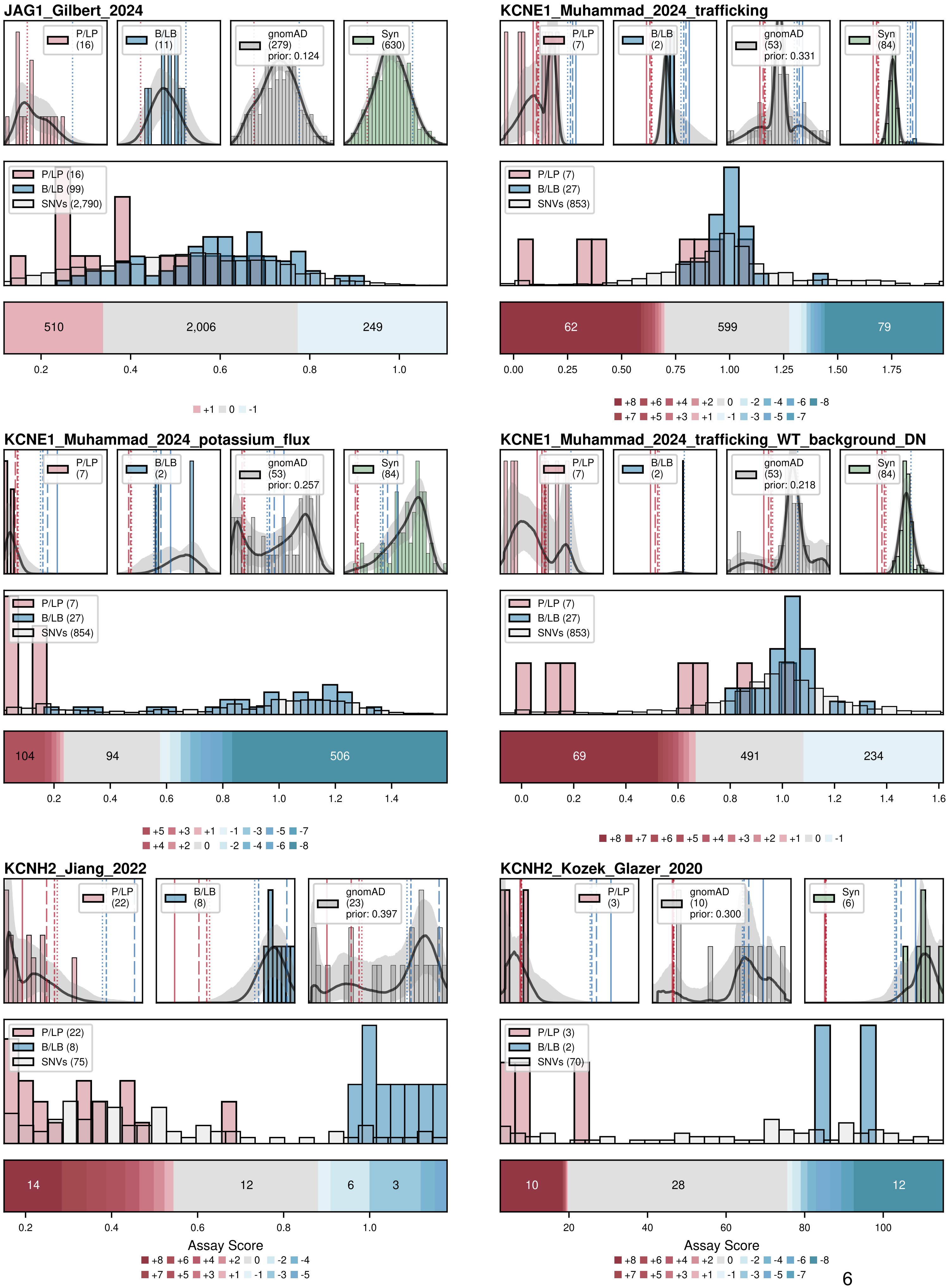

JAG1_Gilbert_2024
P/LP (16)
B/LB (11)
gnomAD (279) prior: 0.124
Syn (630)
P/LP (16)
B/LB (99)
SNVs (2,790)
510
2,006
249
0.2
0.4
0.6
0.8
1.0
+1 0 -1
KCNE1_Muhammad_2024_trafficking
P/LP (7)
B/LB (2)
gnomAD (53) prior: 0.331
Syn (84)
P/LP (7)
B/LB (27)
SNVs (853)
62
599
79
0.00
0.25
0.50
0.75
1.00
1.25
1.50
1.75
+8 +6 +4 +2 0 -2 -4 -6 -8
+7 +5 +3 +1 -1 -3 -5 -7
KCNE1_Muhammad_2024_potassium_flux
P/LP (7)
B/LB (2)
gnomAD (53) prior: 0.257
Syn (84)
P/LP (7)
B/LB (27)
SNVs (854)
104
94
506
0.2
0.4
0.6
0.8
1.0
1.2
1.4
+5 +3 +1 -1 -3 -5 -7
+4 +2 0 -2 -4 -6 -8
KCNE1_Muhammad_2024_trafficking_WT_background_DN
P/LP (7)
B/LB (2)
gnomAD (53) prior: 0.218
Syn (84)
P/LP (7)
B/LB (27)
SNVs (853)
69
491
234
0.0
0.2
0.4
0.6
0.8
1.0
1.2
1.4
1.6
+8 +7 +6 +5 +4 +3 +2 +1 0 -1
KCNH2_Jiang_2022
P/LP (22)
B/LB (8)
gnomAD (23) prior: 0.397
P/LP (22)
B/LB (8)
SNVs (75)
14
12
6
3
0.2
0.4
0.6
0.8
1.0
Assay Score
+8 +6 +4 +2 0 -2 -4
+7 +5 +3 +1 -1 -3 -5
KCNH2_Kozek_Glazer_2020
P/LP (3)
gnomAD (10) prior: 0.300
Syn (6)
P/LP (3)
B/LB (2)
SNVs (70)
10
28
12
20
40
60
80
100
Assay Score
+8 +6 +4 +2 0 -2 -4 -6 -8
+7 +5 +3 +1 -1 -3 -5 -7

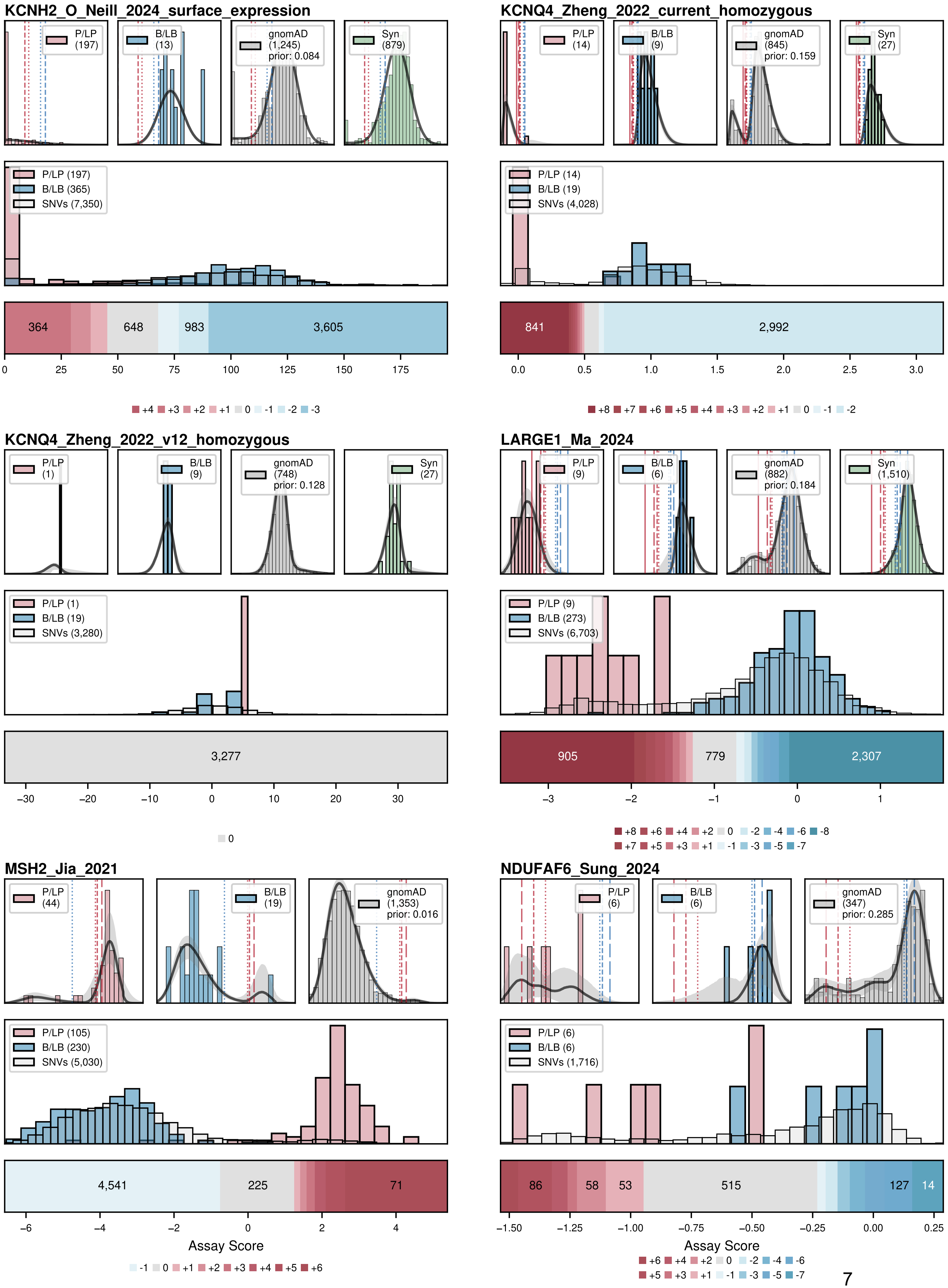
KCNH2_O_Neill_2024_surface_expression
P/LP (197)
B/LB (13)
gnomAD (1,245) prior: 0.084
Syn (879)
P/LP (197)
B/LB (365)
SNVs (7,350)
364
648
983
3,605
0
25
50
75
100
125
150
175
+4 +3 +2 +1 0 -1 -2 -3
KCNQ4_Zheng_2022_current_homozygous
P/LP (14)
B/LB (9)
gnomAD (845) prior: 0.159
Syn (27)
P/LP (14)
B/LB (19)
SNVs (4,028)
841
2,992
0.0
0.5
1.0
1.5
2.0
2.5
3.0
+8 +7 +6 +5 +4 +3 +2 +1 0 -1 -2
KCNQ4_Zheng_2022_v12_homozygous
P/LP (1)
B/LB (9)
gnomAD (748) prior: 0.128
Syn (27)
P/LP (1)
B/LB (19)
SNVs (3,280)
3,277
−30
−20
−10
0
10
20
30
0
LARGE1_Ma_2024
P/LP (9)
B/LB (6)
gnomAD (882) prior: 0.184
Syn (1,510)
P/LP (9)
B/LB (273)
SNVs (6,703)
905
779
2,307
−3
−2
−1
0
1
+8 +6 +4 +2 0 -2 -4 -6 -8
+7 +5 +3 +1 -1 -3 -5 -7
MSH2_Jia_2021
P/LP (44)
B/LB (19)
gnomAD (1,353) prior: 0.016
P/LP (105)
B/LB (230)
SNVs (5,030)
4,541
225
71
−6
−4
−2
0
2
4
Assay Score
-1 0 +1 +2 +3 +4 +5 +6
NDUFAF6_Sung_2024
P/LP (6)
B/LB (6)
gnomAD (347) prior: 0.285
P/LP (6)
B/LB (6)
SNVs (1,716)
86
58
53
515
127
14
−1.50
−1.25
−1.00
−0.75
−0.50
−0.25
0.00
0.25
Assay Score
+6 +4 +2 0 -2 -4 -6
+5 +3 +1 -1 -3 -5 -7

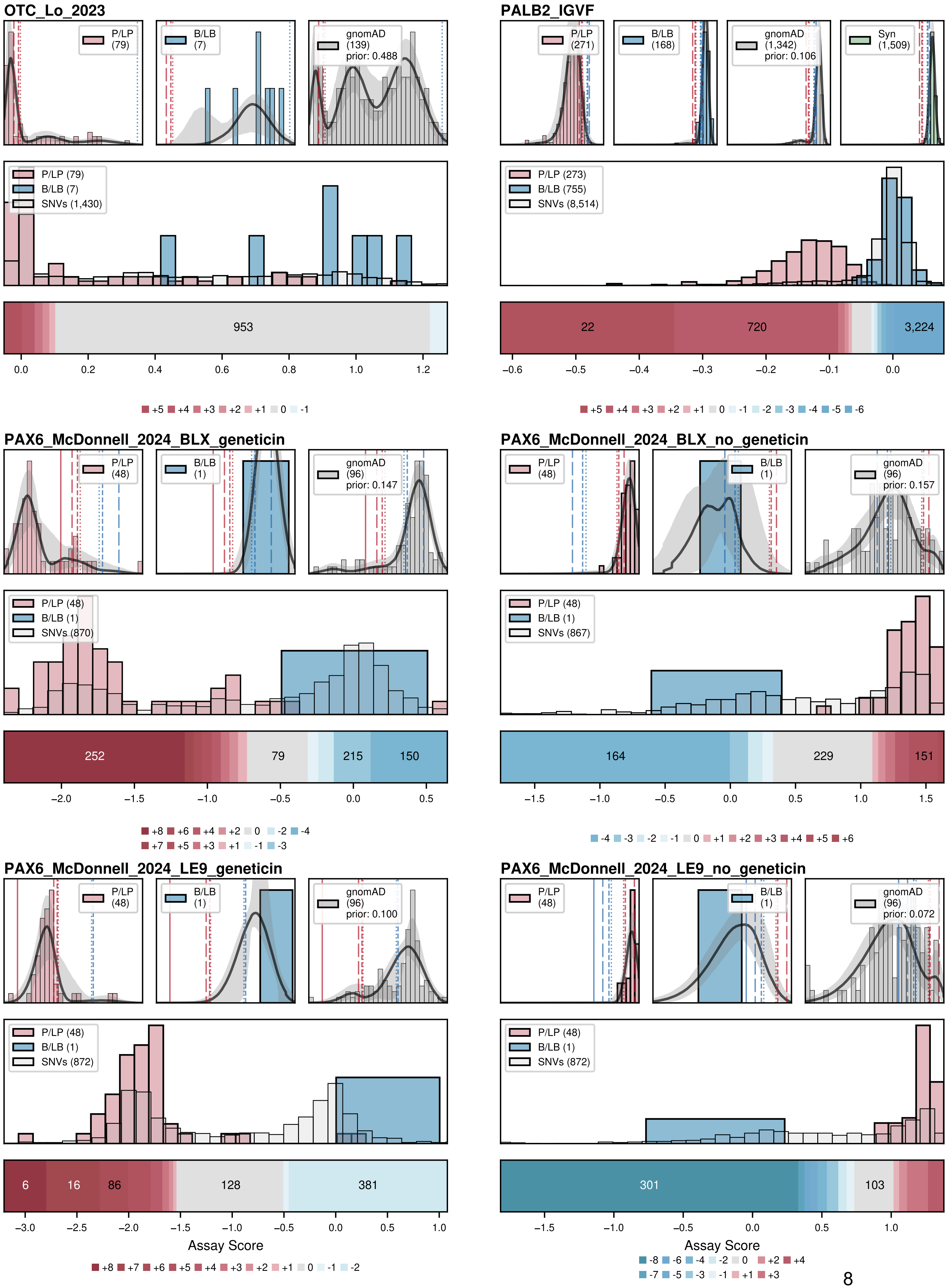

OTC_Lo_2023
P/LP (79)
B/LB (7)
gnomAD (139) prior: 0.488
P/LP (79)
B/LB (7)
SNVs (1,430)
953
0.0 0.2 0.4 0.6 0.8 1.0 1.2
+5 +4 +3 +2 +1 0 -1
PALB2_IGVF
P/LP (271)
B/LB (168)
gnomAD (1,342) prior: 0.106
Syn (1,509)
P/LP (273)
B/LB (755)
SNVs (8,514)
22
720
3,224
−0.6 −0.5 −0.4 −0.3 −0.2 −0.1 0.0
+5 +4 +3 +2 +1 0 -1 -2 -3 -4 -5 -6
PAX6_McDonnell_2024_BLX_geneticin
P/LP (48)
B/LB (1)
gnomAD (96) prior: 0.147
P/LP (48)
B/LB (1)
SNVs (870)
252
79
215
150
−2.0 −1.5 −1.0 −0.5 0.0 0.5
+8 +6 +4 +2 0 -2 -4
+7 +5 +3 +1 -1 -3
PAX6_McDonnell_2024_BLX_no_geneticin
P/LP (48)
B/LB (1)
gnomAD (96) prior: 0.157
P/LP (48)
B/LB (1)
SNVs (867)
164
229
151
−1.5 −1.0 −0.5 0.0 0.5 1.0 1.5
-4 -3 -2 -1 0 +1 +2 +3 +4 +5 +6
PAX6_McDonnell_2024_LE9_geneticin
P/LP (48)
B/LB (1)
gnomAD (96) prior: 0.100
P/LP (48)
B/LB (1)
SNVs (872)
6
16
86
128
381
−3.0 −2.5 −2.0 −1.5 −1.0 −0.5 0.0 0.5 1.0
Assay Score
+8 +7 +6 +5 +4 +3 +2 +1 0 -1 -2
PAX6_McDonnell_2024_LE9_no_geneticin
P/LP (48)
B/LB (1)
gnomAD (96) prior: 0.072
P/LP (48)
B/LB (1)
SNVs (872)
301
103
−1.5 −1.0 −0.5 0.0 0.5 1.0
Assay Score
-8 -6 -4 -2 0 +2 +4
-7 -5 -3 -1 +1 +3

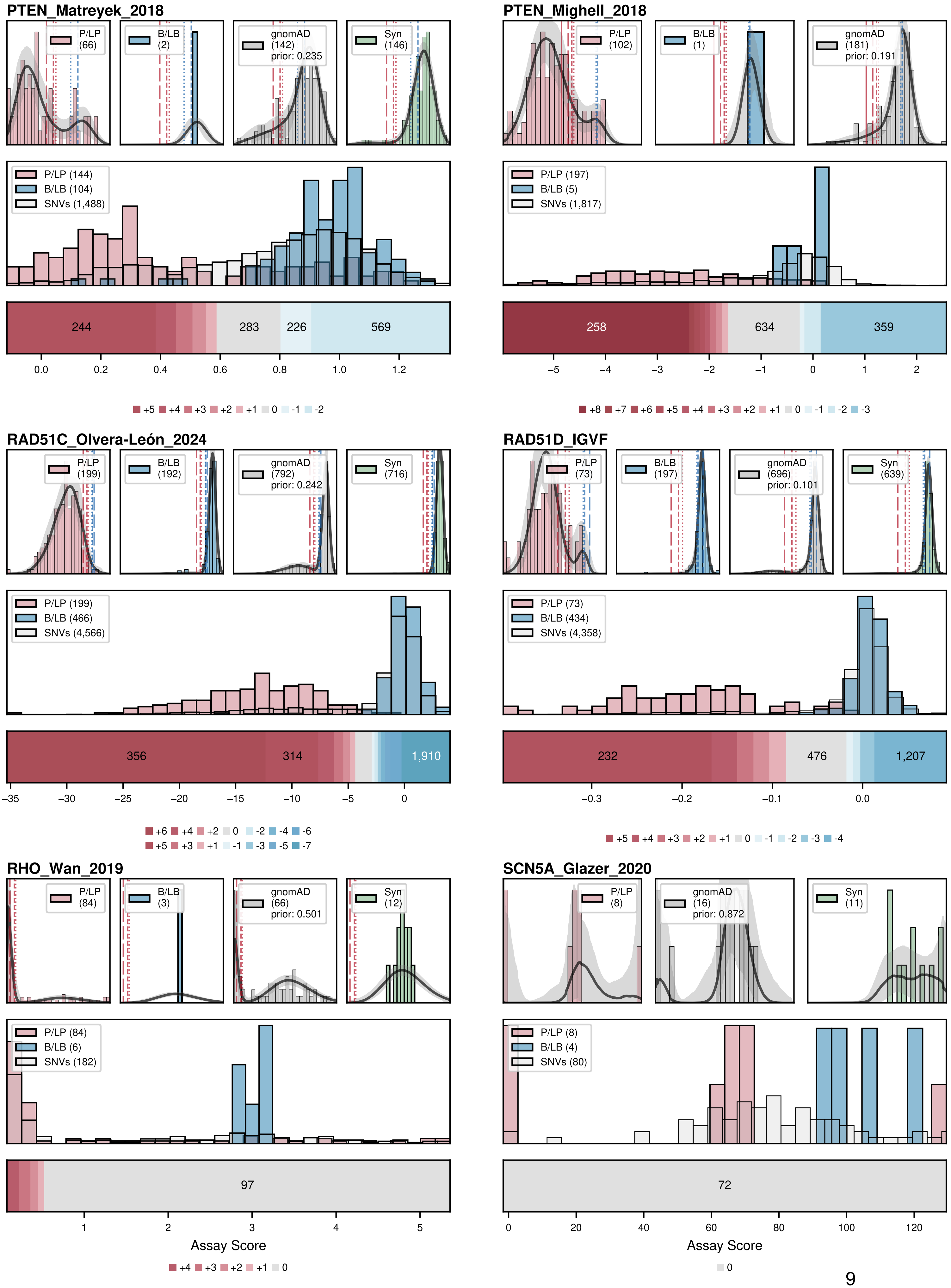
PTEN_Matreyek_2018
P/LP (66)
B/LB (2)
gnomAD (142) prior: 0.235
Syn (146)
P/LP (144)
B/LB (104)
SNVs (1,488)
244
283
226
569
0.0 0.2 0.4 0.6 0.8 1.0 1.2
+5 +4 +3 +2 +1 0 -1 -2
PTEN_Mighell_2018
P/LP (102)
B/LB (1)
gnomAD (181) prior: 0.191
P/LP (197)
B/LB (5)
SNVs (1,817)
258
634
359
−5 −4 −3 −2 −1 0 1 2
+8 +7 +6 +5 +4 +3 +2 +1 0 -1 -2 -3
RAD51C_Olvera-León_2024
P/LP (199)
B/LB (192)
gnomAD (792) prior: 0.242
Syn (716)
P/LP (199)
B/LB (466)
SNVs (4,566)
356
314
1,910
−35 −30 −25 −20 −15 −10 −5 0
+6 +4 +2 0 -2 -4 -6
+5 +3 +1 -1 -3 -5 -7
RAD51D_IGVF
P/LP (73)
B/LB (197)
gnomAD (696) prior: 0.101
Syn (639)
P/LP (73)
B/LB (434)
SNVs (4,358)
232
476
1,207
−0.3 −0.2 −0.1 0.0
+5 +4 +3 +2 +1 0 -1 -2 -3 -4
RHO_Wan_2019
P/LP (84)
B/LB (3)
gnomAD (66) prior: 0.501
Syn (12)
P/LP (84)
B/LB (6)
SNVs (182)
97
1 2 3 4 5
Assay Score
+4 +3 +2 +1 0
SCN5A_Glazer_2020
P/LP (8)
gnomAD (16) prior: 0.872
Syn (11)
P/LP (8)
B/LB (4)
SNVs (80)
72
0 20 40 60 80 100 120
Assay Score
0

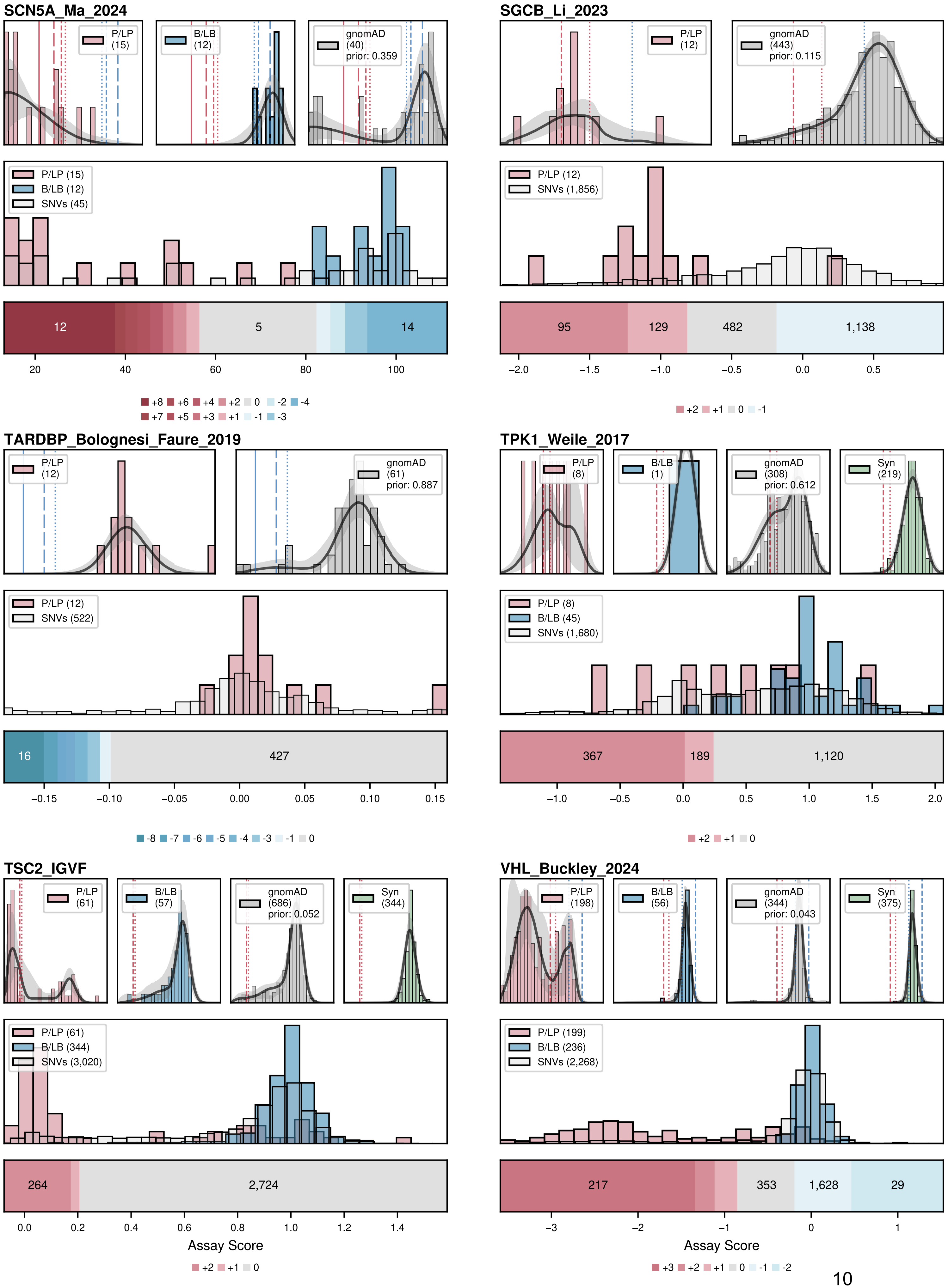
SCN5A_Ma_2024
P/LP (15)
B/LB (12)
gnomAD (40) prior: 0.359
P/LP (15)
B/LB (12)
SNVs (45)
12
5
14
20
40
60
80
100
+8 +6 +4 +2 0 -2 -4
+7 +5 +3 +1 -1 -3
SGCB_Li_2023
P/LP (12)
gnomAD (443) prior: 0.115
P/LP (12)
SNVs (1,856)
95
129
482
1,138
−2.0
−1.5
−1.0
−0.5
0.0
0.5
+2 +1 0 -1
TARDBP_Bolognesi_Faure_2019
P/LP (12)
gnomAD (61) prior: 0.887
P/LP (12)
SNVs (522)
16
427
−0.15
−0.10
−0.05
0.00
0.05
0.10
0.15
-8 -7 -6 -5 -4 -3 -1 0
TPK1_Weile_2017
P/LP (8)
B/LB (1)
gnomAD (308) prior: 0.612
Syn (219)
P/LP (8)
B/LB (45)
SNVs (1,680)
367
189
1,120
−1.0
−0.5
0.0
0.5
1.0
1.5
2.0
+2 +1 0
TSC2_IGVF
P/LP (61)
B/LB (57)
gnomAD (686) prior: 0.052
Syn (344)
P/LP (61)
B/LB (344)
SNVs (3,020)
264
2,724
0.0
0.2
0.4
0.6
0.8
1.0
1.2
1.4
Assay Score
+2 +1 0
VHL_Buckley_2024
P/LP (198)
B/LB (56)
gnomAD (344) prior: 0.043
Syn (375)
P/LP (199)
B/LB (236)
SNVs (2,268)
217
353
1,628
29
−3
−2
−1
0
1
Assay Score
+3 +2 +1 0 -1 -2

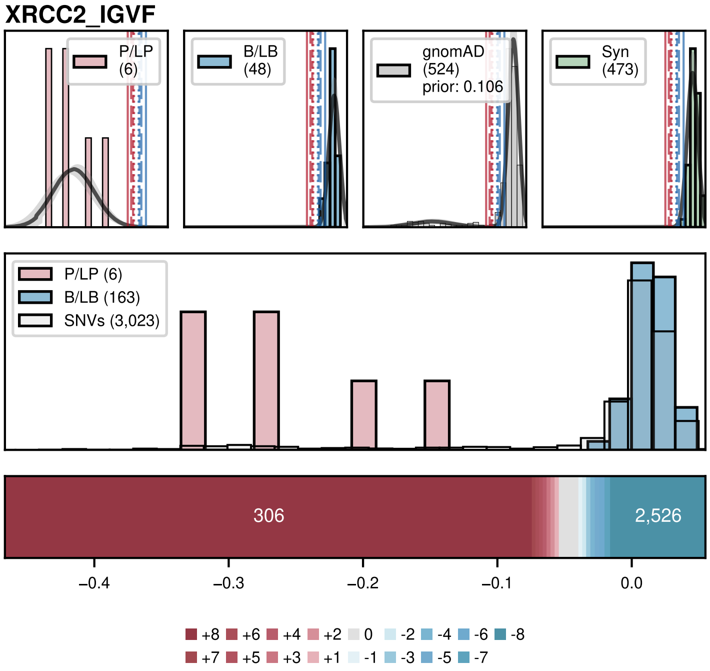

Extended Data Figure 1: ExCALIBR model fits and assigned evidence for experimental datasets

ExCALIBR fits, variant score distributions, and calibrated evidence strengths are shown for 61 experimental datasets spanning 37 genes across 11 pages, with each dataset visualized in a single panel containing three components: top, ExCALIBR fits for pathogenic variants (red), benign variants (blue), gnomAD population variants (grey), and synonymous variants (green; exclusive with other variant classes when available), where ClinVar variants are from the December 2018 release for BRCA1, MSH2, TP53, and PTEN, and the January 2025 release for all other genes; middle, score distributions for pathogenic (red) and benign (blue) control variants from the January 2025 ClinVar release, overlaid with the distribution of all possible SNVs (light grey); bottom, score intervals corresponding to evidence strength assignments (up to 8 pathogenic or benign points) determined by ExCALIBR, with the number of SNVs assigned to each interval labeled within sufficiently large bins.

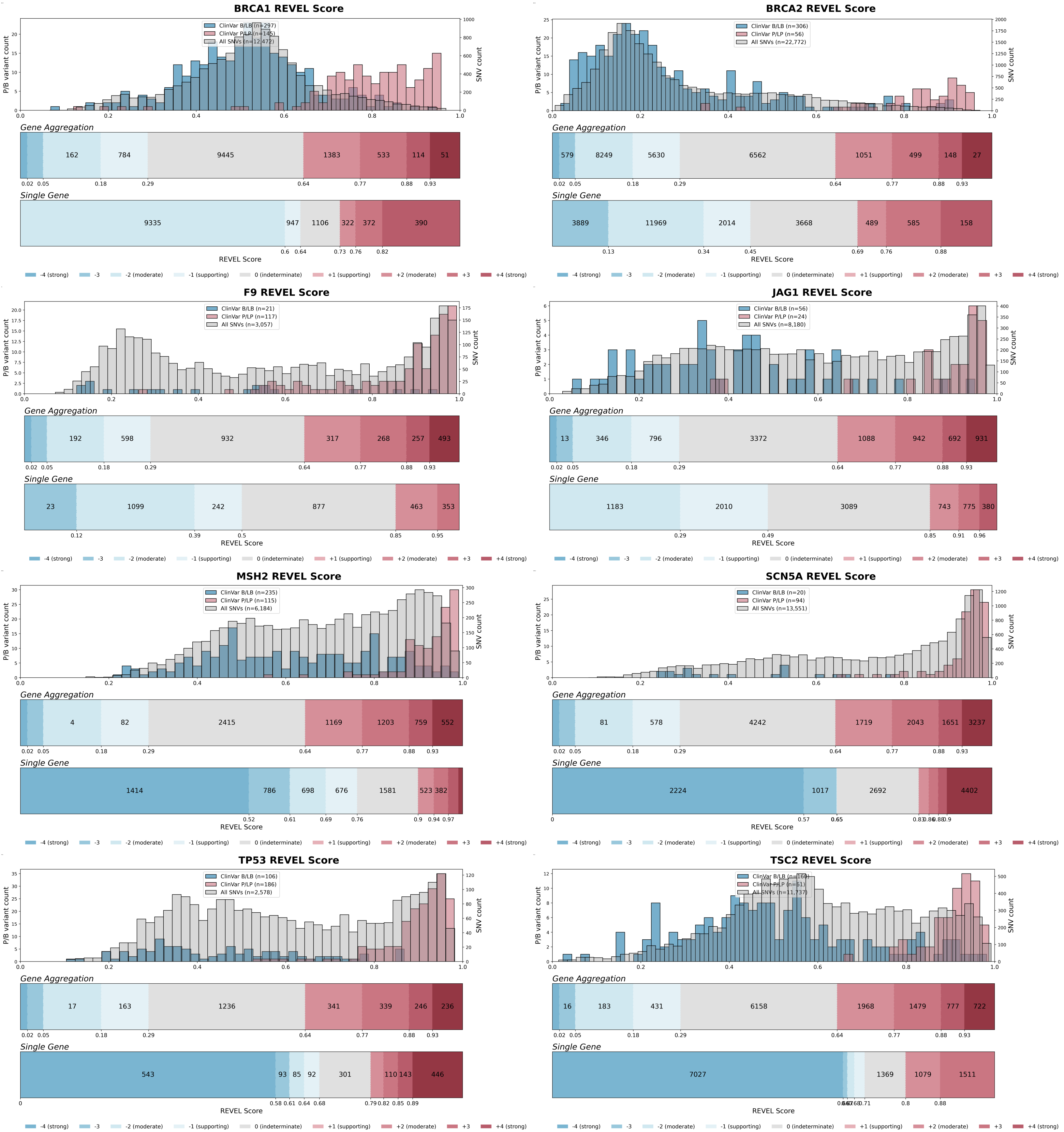

BRCA1 REVEL Score
ClinVar B/LB (n=297)
ClinVar P/LP (n=145)
All SNVs (n=12,472)
P/B variant count
SNV count
Gene Aggregation
162 784 9445 1383 533 114 51
0.02 0.05 0.18 0.29 0.64 0.77 0.88 0.93
Single Gene
9335 947 1106 322 372 390
0.6 0.64 0.73 0.76 0.82
REVEL Score
BRCA2 REVEL Score
ClinVar B/LB (n=306)
ClinVar P/LP (n=56)
All SNVs (n=22,772)
Gene Aggregation
579 8249 5630 6562 1051 499 148 27
0.02 0.05 0.18 0.29 0.64 0.77 0.88 0.93
Single Gene
3889 11969 2014 3668 489 585 158
0.13 0.34 0.45 0.69 0.76 0.88
REVEL Score
F9 REVEL Score
ClinVar B/LB (n=21)
ClinVar P/LP (n=117)
All SNVs (n=3,057)
Gene Aggregation
192 598 932 317 268 257 493
0.02 0.05 0.18 0.29 0.64 0.77 0.88 0.93
Single Gene
23 1099 242 877 463 353
0.12 0.39 0.5 0.85 0.95
REVEL Score
JAG1 REVEL Score
ClinVar B/LB (n=56)
ClinVar P/LP (n=24)
All SNVs (n=8,180)
Gene Aggregation
13 346 796 3372 1088 942 692 931
0.02 0.05 0.18 0.29 0.64 0.77 0.88 0.93
Single Gene
1183 2010 3089 743 775 380
0.29 0.49 0.85 0.91 0.96
REVEL Score
MSH2 REVEL Score
ClinVar B/LB (n=235)
ClinVar P/LP (n=115)
All SNVs (n=6,184)
Gene Aggregation
4 82 2415 1169 1203 759 552
0.02 0.05 0.18 0.29 0.64 0.77 0.88 0.93
Single Gene
1414 786 698 676 1581 523 382
0.52 0.61 0.69 0.76 0.9 0.94 0.97
REVEL Score
SCN5A REVEL Score
ClinVar B/LB (n=20)
ClinVar P/LP (n=94)
All SNVs (n=13,551)
Gene Aggregation
81 578 4242 1719 2043 1651 3237
0.02 0.05 0.18 0.29 0.64 0.77 0.88 0.93
Single Gene
2224 1017 2692 4402
0 0.57 0.65 0.83 0.86 0.88 0.9
REVEL Score
TP53 REVEL Score
ClinVar B/LB (n=106)
ClinVar P/LP (n=186)
All SNVs (n=2,578)
Gene Aggregation
17 163 1236 341 339 246 236
0.02 0.05 0.18 0.29 0.64 0.77 0.88 0.93
Single Gene
543 93 85 92 301 110 143 446
0 0.58 0.61 0.64 0.68 0.79 0.82 0.85 0.89
REVEL Score
TSC2 REVEL Score
ClinVar B/LB (n=160)
ClinVar P/LP (n=51)
All SNVs (n=11,737)
Gene Aggregation
16 183 431 6158 1968 1479 777 722
0.02 0.05 0.18 0.29 0.64 0.77 0.88 0.93
Single Gene
7027 1369 1079 1511
0.71 0.8 0.88
REVEL Score
-4 (strong) -3 -2 (moderate) -1 (supporting) 0 (indeterminate) +1 (supporting) +2 (moderate) +3 +4 (strong)

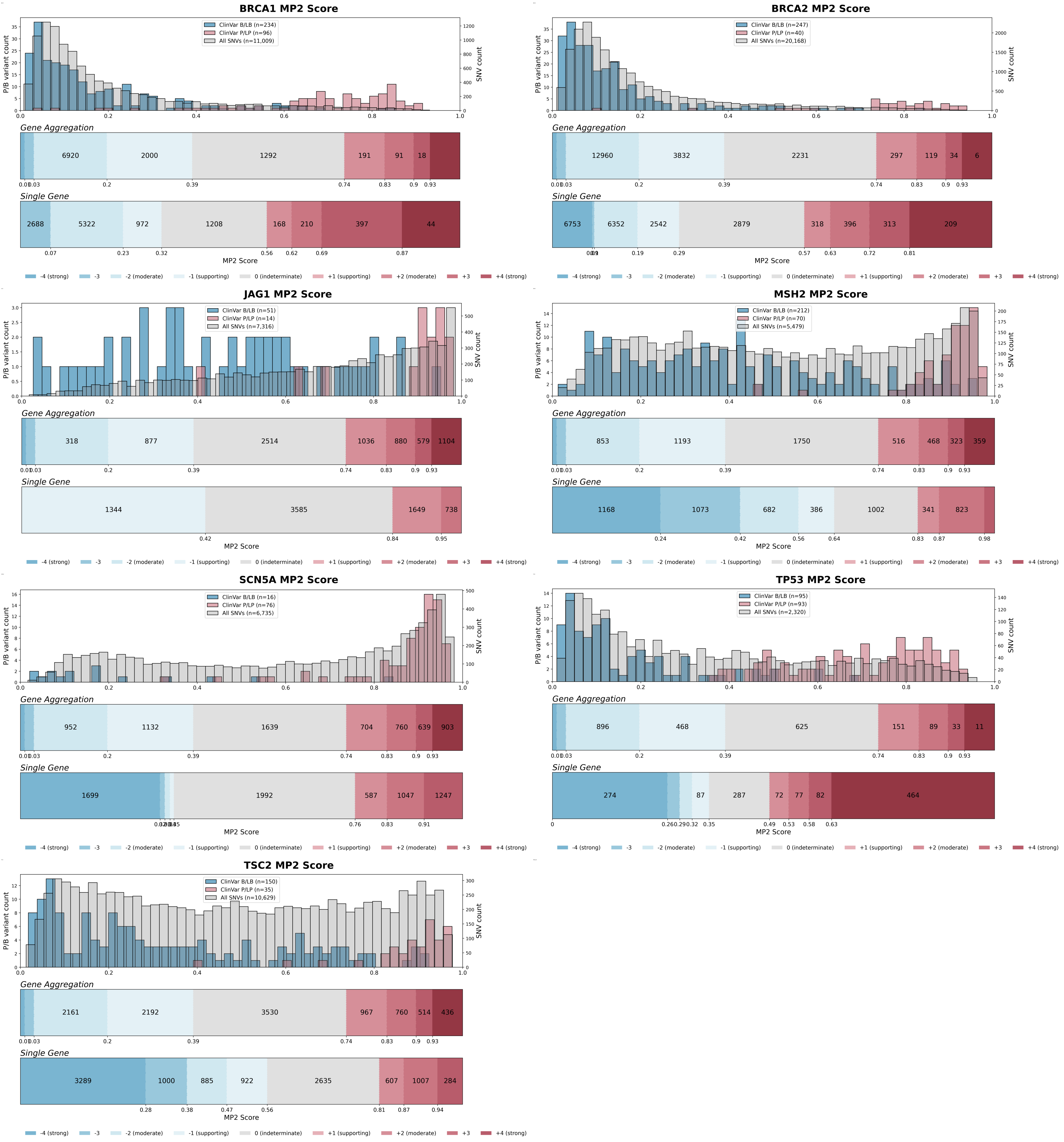
BRCA1 MP2 Score
ClinVar B/LB (n=234)
ClinVar P/LP (n=96)
All SNVs (n=11,009)
P/B variant count
SNV count
Gene Aggregation
6920 2000 1292 191 91 18
0.01 0.03 0.2 0.39 0.74 0.83 0.9 0.93
Single Gene
2688 5322 972 1208 168 210 397 44
0.07 0.23 0.32 0.56 0.62 0.69 0.87
MP2 Score
-4 (strong) -3 -2 (moderate) -1 (supporting) 0 (indeterminate) +1 (supporting) +2 (moderate) +3 +4 (strong)
BRCA2 MP2 Score
ClinVar B/LB (n=247)
ClinVar P/LP (n=40)
All SNVs (n=20,168)
Gene Aggregation
12960 3832 2231 297 119 34 6
Single Gene
6753 6352 2542 2879 318 396 313 209
0.09 0.1 0.19 0.29 0.57 0.63 0.72 0.81
JAG1 MP2 Score
ClinVar B/LB (n=51)
ClinVar P/LP (n=14)
All SNVs (n=7,316)
Gene Aggregation
318 877 2514 1036 880 579 1104
Single Gene
1344 3585 1649 738
0.42 0.84 0.95
MSH2 MP2 Score
ClinVar B/LB (n=212)
ClinVar P/LP (n=70)
All SNVs (n=5,479)
Gene Aggregation
853 1193 1750 516 468 323 359
Single Gene
1168 1073 682 386 1002 341 823
0.24 0.42 0.56 0.64 0.83 0.87 0.98
SCN5A MP2 Score
ClinVar B/LB (n=16)
ClinVar P/LP (n=76)
All SNVs (n=6,735)
Gene Aggregation
952 1132 1639 704 760 639 903
Single Gene
1699 1992 587 1047 1247
0.76 0.83 0.91
TP53 MP2 Score
ClinVar B/LB (n=95)
ClinVar P/LP (n=93)
All SNVs (n=2,320)
Gene Aggregation
896 468 625 151 89 33 11
Single Gene
274 87 287 72 77 82 464
0 0.26 0.29 0.32 0.35 0.49 0.53 0.58 0.63
TSC2 MP2 Score
ClinVar B/LB (n=150)
ClinVar P/LP (n=35)
All SNVs (n=10,629)
Gene Aggregation
2161 2192 3530 967 760 514 436
Single Gene
3289 1000 885 922 2635 607 1007 284
0.28 0.38 0.47 0.56 0.81 0.87 0.94

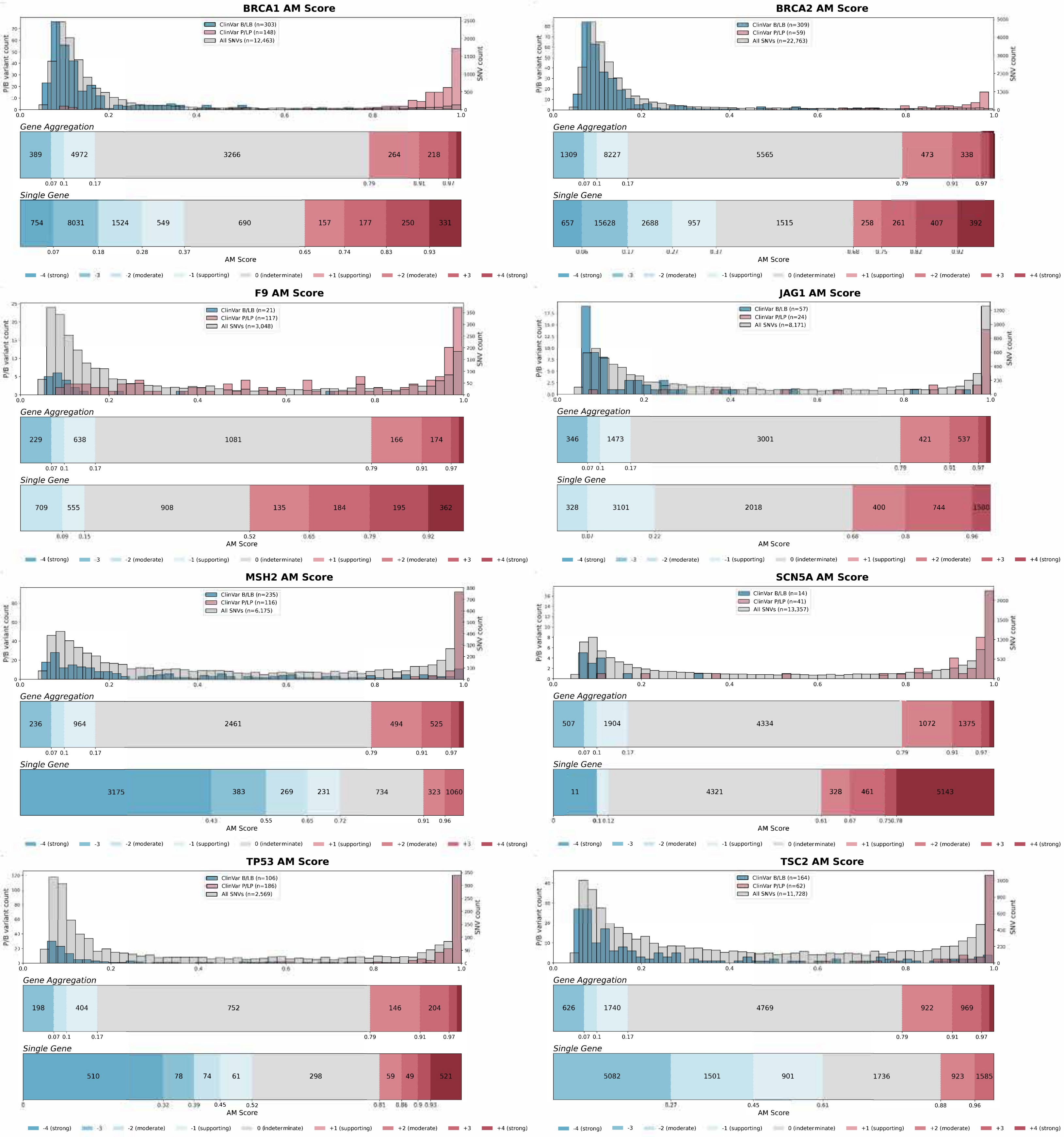

BRCA1 AM Score
ClinVar B/LB (n=303)
ClinVar P/LP (n=148)
All SNVs (n=12,463)
P/B variant count
SNV count
Gene Aggregation
389
4972
3266
264
218
Single Gene
754
8031
1524
549
690
157
177
250
331
AM Score
BRCA2 AM Score
ClinVar B/LB (n=309)
ClinVar P/LP (n=59)
All SNVs (n=22,763)
Gene Aggregation
1309
8227
5565
473
338
Single Gene
657
15628
2688
957
1515
258
261
407
392
F9 AM Score
ClinVar B/LB (n=21)
ClinVar P/LP (n=117)
All SNVs (n=3,048)
Gene Aggregation
229
638
1081
166
174
Single Gene
709
555
908
135
184
195
362
JAG1 AM Score
ClinVar B/LB (n=57)
ClinVar P/LP (n=24)
All SNVs (n=8,171)
Gene Aggregation
346
1473
3001
421
537
Single Gene
328
3101
2018
400
744
1580
MSH2 AM Score
ClinVar B/LB (n=235)
ClinVar P/LP (n=116)
All SNVs (n=6,175)
Gene Aggregation
236
964
2461
494
525
Single Gene
3175
383
269
231
734
323
1060
SCN5A AM Score
ClinVar B/LB (n=14)
ClinVar P/LP (n=41)
All SNVs (n=13,357)
Gene Aggregation
507
1904
4334
1072
1375
Single Gene
11
4321
328
461
5143
TP53 AM Score
ClinVar B/LB (n=106)
ClinVar P/LP (n=186)
All SNVs (n=2,569)
Gene Aggregation
198
404
752
146
204
Single Gene
510
78
74
61
298
59
49
521
TSC2 AM Score
ClinVar B/LB (n=164)
ClinVar P/LP (n=62)
All SNVs (n=11,728)
Gene Aggregation
626
1740
4769
922
969
Single Gene
5082
1501
901
1736
923
1585
-4 (strong)
-3
-2 (moderate)
-1 (supporting)
0 (indeterminate)
+1 (supporting)
+2 (moderate)
+3
+4 (strong)

Extended Data Figure 3: Gene-specific predictor calibrations across 8 genes for REVEL, MutPred2, and AlphaMissense

Gene-specific calibrations for REVEL, MutPred2, and AlphaMissense, respectively, across 8 genes, each visualized with three components: top, pathogenic (red) and benign (blue) control missense variants from the ClinVar January 2025 release overlaid with all possible missense SNVs (light grey); middle, genome-wide aggregation score thresholds; bottom, gene-specific calibration score intervals, with both middle and bottom panels labeled by evidence strength (up to 4 pathogenic or benign points) and SNV counts shown within sufficiently large bins. Calibrations of MutPred2 scores for F9 are not shown because after excluding variants used in MutPred2 training, the remaining control variants were insufficient to support a robust gene-specific calibration.
