# Supplementary material for: A scalable approach to resolving variants of uncertain significance
Source: bioRxiv. 2026 Feb 23:2026.02.14.705848. Originally published 2026 Feb 14. Preprint. [Version 2] doi: 10.64898/2026.02.14.705848 (PMC12918978; doi:10.64898/2026.02.14.705848)
Supplement: 1 [file NIHPP2026.02.14.705848V2-supplement-1.pdf]

bioRxiv preprint doi: https://doi.org/10.64898/2026.02.14.705848; this version posted February 23, 2026. The copyright holder for this preprint (which was not certified by peer review) is the author/funder, who has granted bioRxiv a license to display the preprint in perpetuity. It is made available under aCC-BY 4.0 International license.

Supplementary Notes

Supplementary Note 1

PTEN Variant Misclassification

One misclassified variant was *PTEN* (NM_000314.6:c.500C>A), a *de novo* variant associated with autism spectrum disorder. Experimental evidence supported a benign classification (−2 points) while predictive evidence supported pathogenicity (+1 point), resulting in −1 combined points and an LB classification. This discordance suggests that the experimental assay measuring lipid phosphatase activity[108] may not fully capture the ASD-relevant mechanism for PTEN. Specifically, NM_000314.8(PTEN):c.500C>A (p.Thr167Asn) was identified *de novo* in a 99-month-old white female with autism spectrum disorder from the Simons Simplex Collection (SSC; proband 11390.p1)[109]. The individual presented with macrocephaly, speech delay with regression, social communication deficits, ADHD, pica, sleep disturbances, recurrent otitis media, and gastrointestinal issues. Family history is notable for autism features in the mother, ADHD in a sibling, and an extensive family history of neuropsychiatric conditions. Both parents and the sibling did not have macrocephaly. While functional evidence does not show that this variant is low abundance or lacking lipid phosphatase activity, we have recently learned PTEN variants associated with neurodevelopmental disorders may disrupt PTEN function through other mechanisms. A recent study which used visual phenotype to assess variant effects on PTEN subcellular localization demonstrated that this variant is nuclearly mislocalized, similar to other PTEN variants known to be associated with neurodevelopmental disorders[91].

Supplementary Note 2

Minimum Sensitivity for BS3_supporting

The likelihood ratio for a normal assay result (LR−) equals (1 − sensitivity) divided by specificity. Setting LR− equal to the BS3_supporting threshold of 0.48 and solving for sensitivity at 90% specificity yields a minimum sensitivity of 56.8%, calculated as 1 − (0.48 × 0.90). An assay with 90% specificity can therefore have sensitivity as low as 57% and still qualify for BS3_supporting, meaning such an assay fails to detect 43% of pathogenic variants.

bioRxiv preprint doi: https://doi.org/10.64898/2026.02.14.705848; this version posted February 23, 2026. The copyright holder for this preprint (which was not certified by peer review) is the author/funder, who has granted bioRxiv a license to display the preprint in perpetuity. It is made available under aCC-BY 4.0 International license.

False Omission Rate at Different Priors

The false omission rate (FOR) equals the product of (1 − sensitivity) and the prior probability, divided by the sum of that product and the product of specificity and (1 − prior). At 57% sensitivity and 90% specificity, the FOR is 4.5% when the prior probability of pathogenicity is 10%, rises to 13.7% at a 25% prior, and reaches 32.3% at a 50% prior. When the prior exceeds the ACMG/AMP assumption of 10%, the proportion of misclassified pathogenic variants increases substantially. At a 50% prior, nearly one in three likely benign classifications based on single-source weak evidence would be incorrect.

bioRxiv preprint doi: https://doi.org/10.64898/2026.02.14.705848; this version posted February 23, 2026. The copyright holder for this preprint (which was not certified by peer review) is the author/funder, who has granted bioRxiv a license to display the preprint in perpetuity. It is made available under aCC-BY 4.0 International license.

Supplementary Tables

Supplementary Table 1: Table listing IGVF accession numbers and corresponding MaveDB URNs for access to raw variant effect measurements and functional calibration data used in this analysis.

bioRxiv preprint doi: https://doi.org/10.64898/2026.02.14.705848; this version posted February 23, 2026. The copyright holder for this preprint (which was not certified by peer review) is the author/funder, who has granted bioRxiv a license to display the preprint in perpetuity. It is made available under aCC-BY 4.0 International license.

Name	Analysis Set Accession	Gene	Assay	URL	MaveDB urn
BRCA2 SGE scores (BRCA2_IGVF)	IGVFDS4107SKOE	BRCA2	Saturation Genome Editing	data.igvf.org/analysis-sets/IGVFDS4107SKOE	urn:mavedb:00001263-a-1
RAD51D SGE scores (RAD51D_IGVF)	IGVFDS0913XYYZ	RAD51D	Saturation Genome Editing	data.igvf.org/analysis-sets/IGVFDS0913XYYZ	urn:mavedb:00001260-a-1
BARD1 SGE scores (BARD1_IGVF)	IGVFDS4537CHCJ	BARD1	Saturation Genome Editing	data.igvf.org/analysis-sets/IGVFDS4537CHCJ	urn:mavedb:00001250-a-1
PALB2 SGE scores (PALB2_IGVF)	IGVFDS4890LMRX	PALB2	Saturation Genome Editing	data.igvf.org/analysis-sets/IGVFDS4890LMRX	urn:mavedb:00001259-a-1
CTCF SGE scores (CTCF_IGVF)	IGVFDS5747FHZI	CTCF	Saturation Genome Editing	data.igvf.org/analysis-sets/IGVFDS5747FHZI	urn:mavedb:00001262-a-1
XRCC2 SGE scores (XRCC2_IGVF)	IGVFDS1568IEJQ	XRCC2	Saturation Genome Editing	data.igvf.org/analysis-sets/IGVFDS1568IEJQ	urn:mavedb:00001264-a-1
SFPQ SGE scores (SFPQ_IGVF)	IGVFDS2617CLAY	SFPQ	Saturation Genome Editing	data.igvf.org/analysis-sets/IGVFDS2617CLAY	urn:mavedb:00001265-a-1
G6PD DMS scores (G6PD_IGVF)	IGVFDS1322SJZB	G6PD	VAMP-seq	data.igvf.org/analysis-sets/IGVFDS1322SJZB	urn:mavedb:00001266-b-1
TSC2 DMS scores (Tuberin, RAP-GAP) (TSC2_IGVF)	IGVFDS5595BTYJ	TSC2	VAMP-seq	data.igvf.org/analysis-sets/IGVFDS5595BTYJ	urn:mavedb:00001267-b-1
F9 Carboxylation F9-Gla scores (F9_Popp_2025_carboxy_F9_specific)	IGVFDS8157CREJ	F9	VAMP-seq (MultiSTEP)	data.igvf.org/analysis-sets/IGVFDS8157CREJ	urn:mavedb:00001200-d-1
F9 Carboxylation pan-Gla scores (F9_Popp_2025_carboxy_gla_motif)	IGVFDS8560WCPN	F9	VAMP-seq (MultiSTEP)	data.igvf.org/analysis-sets/IGVFDS8560WCPN	urn:mavedb:00001200-e-1
F9 Strep II tag antibody scores (F9_Popp_2025_strep_2	IGVFDS7535YOJR	F9	VAMP-seq (MultiSTEP)	data.igvf.org/analysis-sets/IGVFDS7535YOJR	urn:mavedb:00001200-a-1

bioRxiv preprint doi: https://doi.org/10.64898/2026.02.14.705848; this version posted February 23, 2026. The copyright holder for this preprint (which was not certified by peer review) is the author/funder, who has granted bioRxiv a license to display the preprint in perpetuity. It is made available under aCC-BY 4.0 International license.

)					
F9 light chain antibody scores (F9_Popp_2025_light_chain)	IGVFDS4868IDCK	F9	VAMP-seq (MultiSTEP)	data.igvf.org/analysis-sets/IGVFDS4868IDCK	urn:mavedb:00001200-c-1
F9 heavy chain antibody scores (F9_Popp_2025_heavy_chain)	IGVFDS8821RPWB	F9	VAMP-seq (MultiSTEP)	data.igvf.org/analysis-sets/IGVFDS8821RPWB	urn:mavedb:00001200-b-1
Abundance by DUAL-IPA	IGVFDS0761PJSK	Multiple	DUAL-IPA	data.igvf.org/analysis-sets/IGVFDS0761PJSK	
Edgotyping by sqY2H	IGVFDS9876GIEQ	Multiple	Yeast Two-Hybrid	data.igvf.org/analysis-sets/IGVFDS9876GIEQ	
Mislocalization by variant painting	IGVFDS4288KLWA	Multiple	Variant Painting	data.igvf.org/analysis-sets/IGVFDS4288KLWA	
Integrated variant effect dataset	IGVFFI2521UGYG	Multiple	Multiple	data.igvf.org/curated-sets/IGVFDS4011IBUI	
Biobank validation of variant classifications	IGVFDS5836NKME	Multiple	NA	data.igvf.org/tabular-files/IGVFFI3804AVJR	
ExCALIBR assay calibration thresholds	IGVFFI5038ZNHZ	Multiple	Multiple	data.igvf.org/tabular-files/IGVFFI5038ZNHZ	
ExCALIBR per variant evidence points	IGVFFI7610PCPU	Multiple	Multiple	data.igvf.org/tabular-files/IGVFFI7610PCPU	
ExCALIBR and gene-specific per variant evidence points	IGVFFI1443TQDN	Multiple	Multiple	data.igvf.org/tabular-files/IGVFFI1443TQDN	

bioRxiv preprint doi: https://doi.org/10.64898/2026.02.14.705848; this version posted February 23, 2026. The copyright holder for this preprint (which was not certified by peer review) is the author/funder, who has granted bioRxiv a license to display the preprint in perpetuity. It is made available under aCC-BY 4.0 International license.

Supplementary Table 2: Table of gene-disease pairs that were excluded from biobank validation, detailing for each pair whether it was excluded because the mode of inheritance is not autosomal dominant, due to the absence of a matching phenotype in AoU, or due to a lack of participants with the phenotype and variants in the gene.

**Gene**	**Associated phenotype(s)**	**Not autosomal dominant**	**No matching Condition in AoU**	**Fewer than 40 cases with variants in gene**
ASPA	Canavan disease	✘	✘	
CALM1/2/3	Long QT syndrome or Sudden cardiac death			✘
CARD11	immunodeficiency 11b with atopic dermatitis, BENTA disease		✘	
CBS	Classic homocystinuria	✘		✘
CRX	Cone-rod dystrophy			✘
CTCF	Syndromic intellectual disability			✘
DDX3X	X-linked syndromic intellectual disability	✘		✘
F9	Hemophilia B	✘		✘
FKRP	Myopathy caused by variation in FKRP	✘	✘	
G6PD	Deficiency of glucose-6-phosphate dehydrogenase	✘		
HMBS	Acute intermittent porphyria	✘		✘
JAG1	Alagille syndrome/Arteriohepatic dysplasia		✘	
LARGE1	Dystroglycanopathy	✘	✘	
NDUFAF6	Leigh's disease	✘		✘
OTC	Ornithine carbamoyltransferase deficiency	✘	✘	
PAX6	Ocular dysgenesis		✘	
RHO	Retinitis pigmentosa	✘		✘
SFPQ	Renal cell carcinoma or Bone sarcoma			✘
SGCB	Limb-girdle muscular dystrophy	✘		✘
TARDBP	Amyotrophic lateral sclerosis or Frontotemporal dementia			✘
TPK1	Leigh's disease	✘		✘

bioRxiv preprint doi: https://doi.org/10.64898/2026.02.14.705848; this version posted February 23, 2026. The copyright holder for this preprint (which was not certified by peer review) is the author/funder, who has granted bioRxiv a license to display the preprint in perpetuity. It is made available under aCC-BY 4.0 International license.

VHL	von Hippel-Lindau disease			✘
XRCC2	Fanconi anemia	✘		✘

Supplementary Table 3: Table of gene-disease pairs detailing for each gene the criteria for including participants in the gene's case cohort and the criteria for excluding participants from the gene's control cohort.

**Gene**	**Case Inclusion Criteria**	**Control Exclusion Criteria**
BAP1	Melanoma, Mesothelioma, Renal cancer, Basal cell carcinoma, Intracranial meningioma, or Choloangiocarcinoma of the biliary tract	Cancer
BARD1	Breast cancer or Ovarian cancer	Cancer
BRCA1	Breast cancer, Ovarian cancer, Prostate cancer, Pancreatic cancer, or Melanoma	Cancer
BRCA2	Breast cancer, Ovarian cancer, Prostate cancer, Pancreatic cancer, or Melanoma	Cancer
CHEK2	Breast cancer or Prostate cancer	Cancer
GCK	Maturity-onset diabetes of the young (MODY)	Diabetes
KCNE1	Long QT syndrome or Sudden cardiac death	Abnormal QT interval, Cardiac arrhythmia, or Sudden cardiac death
KCNH2	Long QT syndrome or Sudden cardiac death	Abnormal QT interval, Cardiac arrhythmia, or Sudden cardiac death
KCNQ4	Nonsyndromic genetic hearing loss	Hearing loss
MSH2	Colorectal cancer, Ovarian cancer, Stomach cancer, Small bowel cancer, Urinary tract cancer, Prostate cancer, Brain cancer, or Endometrial cancer	Cancer
PALB2	Breast cancer, Ovarian cancer, Pancreatic cancer, or Prostate cancer	Cancer
PTEN	Breast cancer, Endometrial cancer, Thyroid cancer, Colorectal cancer, Renal cancer, or Melanoma	Cancer
RAD51C	Breast cancer or Ovarian cancer	Cancer

bioRxiv preprint doi: https://doi.org/10.64898/2026.02.14.705848; this version posted February 23, 2026. The copyright holder for this preprint (which was not certified by peer review) is the author/funder, who has granted bioRxiv a license to display the preprint in perpetuity. It is made available under aCC-BY 4.0 International license.

RAD51D	Breast cancer or Ovarian cancer	Cancer
SCN5A	Long QT syndrome, Dilated cardiomyopathy, or Sudden cardiac death	Abnormal QT interval, Cardiomyopathy, Cardiac arrhythmia, or Sudden cardiac death
TP53	Cancer	Cancer
TSC2	Tuberous sclerosis	Tuberous sclerosis or Cancer
